# TB/HIV co-infection in homelessness and factors associated with loss to follow-up of tuberculosis treatment: a retrospective cohort

**DOI:** 10.1186/s12879-025-11532-y

**Published:** 2025-09-26

**Authors:** Willie Otavio Bueno Bernardi, Stephanie Ribeiro, Rachel Russo Leite, Ricardo Alexandre Arcêncio, Jeanne Marie Rodrigues Stacciarini, Hugo Fernandes, Paula Hino

**Affiliations:** 1 Ribeirão Preto College of Nursing, University of São Paulo, Ribeirão Preto, São Paulo Brazil; 2 Paulista School of Nursing, Federal University of São Paulo Universidade Federal de São Paulo, São Paulo, São Paulo Brazil; 3 São Paulo Municipal Health Department, Health Surveillance Coordination, São Paulo, São Paulo Brazil; 4 School of Nursing, University of Michigan, Ann Arbour, MI United States of America

**Keywords:** Tuberculosis, Coinfection, HIV, Vulnerable populations, Treatment outcome

## Abstract

**Introduction:**

In Brazil, the homeless population is 56 times more likely to become ill with tuberculosis than the general population, plus co-infection with HIV, which further increases this risk, including unfavorable outcomes. São Paulo is a major urban center in the Global South with a large population of people experiencing homelessness, particularly those coinfected with HIV, which poses challenges to clinical care and municipal management. Although it represents a significant public health issue, it remains underexplored in scientific literature. Therefore, this study aims to identify factors associated with loss to follow-up in tuberculosis treatment among homeless co-infected with HIV in São Paulo, Brazil, between 2015 and 2023.

**Method:**

This is a retrospective cohort of TB/HIV co-infection cases in the homeless population of the municipality of São Paulo, Brazil between 2015 and 2023. The data was obtained from the Information System for Diseases and Notification. Descriptive analysis was carried out to characterize the clinical and sociodemographic profile of notified cases and binary logistic regression to identify associated factors.

**Results:**

The results showed a reduction in the percentage of cures and an increase in the loss to follow-up of tuberculosis treatment in the homeless population. Loss to follow-up was associated with the absence of Directly Observed Treatment (ORa = 13.47; 95%CI = 6.17–29.42), positive Sputum smear result at diagnosis (ORa = 3.44; 95%CI = 1.53–7.71) and re-entry after loss to follow-up (ORa = 2.10; 95%CI = 1.12–3.96). The progressive performance of control sputum smear microscopies was considered a protective factor (ORa = 0.52; 95%CI = 0.44–0.61).

**Conclusion:**

The factors associated with the loss of tuberculosis follow-up among the homeless population living with HIV were: type of entry, diagnostic and control bacilloscopy and the use of Directly Observed Treatment, which are mainly derived from health care and the link with the user. Thus, strengthening services and specific supervision strategies, such as the Street Clinic, is essential for controlling TB/HIV co-infection in this population.

**Supplementary Information:**

The online version contains supplementary material available at 10.1186/s12879-025-11532-y.

## Introduction

Tuberculosis (TB) is a disease that has historically been known for being a public health problem, marked by the agreement of targets for its elimination with the Sustainable Development Goals and the EndTB Strategy [1]. Causing around 1.25 million deaths worldwide in 2023, it was considered the leading cause of death from a single infectious agent in the world, second only during the Covid-19 pandemic. TB mainly affects low- and middle-income countries, including Brazil, which has been categorized among the 30 countries with the highest TB and TB/HIV burden in the world [2].

TB and poverty are closely related, and it is considered a socially determined disease because it is associated with issues of undernutrition, hygiene, lack of access to health services, gender, advanced age, housing and dependence on tobacco, alcohol and illicit drug use [3, 4]. Overlapping comorbidities, such as HIV/AIDS, increase the vulnerability of people affected by TB to illness and the unfavorable outcomes of treatment [3–7].

The homeless population, in turn, is a marginalized and socially vulnerable population group that suffers from social stigma and difficulty in accessing healthcare [3, 8–10]. It is a complex phenomenon faced in medium and large urban centers that transcends the sphere of health, involving social issues that are subjective to the individual [11, 12].

TB manifests itself more frequently in the homeless, given some of the factors related to the way homeless people live, such as illicit drug use, tobacco and alcohol abuse, undernutrition, a history of incarceration, immigration, higher rates of TB/HIV co-infection and mental disorders [3, 8–10, 13]. In addition, high indicators of loss to follow-up, treatment incompleteness, resistant TB and low adherence to treatment are observed among homeless when compared to the general population [8, 10, 14–16], which makes it difficult to control the disease and highlights social inequalities, highlighting problems in the management and organization of the health system to tackle the problem [10, 14].

The municipality of São Paulo is considered Brazil’s largest urban center and ranks fifth among the ten largest in the world. It was home to 24.8% of the national homeless population in July 2023, or approximately 54,800 homeless people in the city [17–19]. In 2022, it is estimated that around 480 new cases of TB were reported in the city of São Paulo, reaching an incidence coefficient of 1,841 new cases/100,000 homeless inhabitants, 14.5% of which had TB/HIV co-infection [20], which is evident as a public health problem.

Although the literature shows the outcomes of TB/HIV co-infection, it was observed that the studies were predominantly carried out in developed countries or national context and did not specifically cut out co-infection in the homeless, which limits our understanding of the situation or in low and middle-income countries among this population, especially in a socially and spatially heterogeneous megalopolis like the municipality of São Paulo [8, 14–16, 21, 22]. Thus, this study aims to identify factors associated with loss to follow-up in tuberculosis treatment among homeless individuals co-infected with HIV in São Paulo, Brazil, between 2015 and 2023.

## Methods

### Study design

This is a descriptive-analytical retrospective cohort study conducted among homeless people with TB/HIV co-infection. The research was guided by the tools Strengthening the Reporting of Observational Studies in Epidemiology - STROBE and Reporting of studies Conducted using Observational Routinely-collected health Data - RECORD [23, 24].

### Study setting

The study carried out in the municipality of São Paulo, which has 1,521.2 km² of territory and a population density of 7,528.26 inhabitants/km2 [17]. It is the municipality with the largest number of inhabitants in Brazil, with an estimated population of over 11.4 million, and a Human Development Index of 0.805 [25]. In a 2021 census carried out in the city of São Paulo by the Secretariat of Social Assistance and Development, this figure was 31,884 people [26].

The municipality of São Paulo has a care network with primary care coverage of 71.2% and a Family Health Strategy of 47.9%, as well as a Municipal Network Specialized in Sexually Transmitted Infection/AIDS, which includes Specialized Care Services, among other services [27–29].

For homeless, there is also the Street Clinic, a national strategy linked to Primary Health Care for comprehensive health care for this population, made up of multi-professional teams that work itinerantly in the territory offering health care, such as active search, diagnosis and follow-up for TB [30]. By 2024, there will be 40 Street Clinic teams distributed throughout the municipality of São Paulo, offering comprehensive care to the homeless population [31].

The Specialized Municipal Network has 17 units called Specialized Assistance Services throughout the city of São Paulo, where people with TB/HIV co-infection are cared for, whether they are homeless or not. TB treatment for HIV infection is carried out by primary care, health units or street clinics, a support network, such as cooperative Directly Observed Treatment (DOT). In the homeless, DOT is carried out by professionals from the Street Clinic and may also include the administration of Antiretroviral Therapy (ART).

### Participants

TB notifications finished as loss to follow-up and present HIV co-infection in homeless people notified between 2015 and 2023 in the municipality of São Paulo were considered.

The criteria established for inclusion in the study population were: homeless people with TB/HIV co-infection, aged 15 or over and whose case was closed as “loss to follow-up” or “primary loss to follow-up”. In the Brazilian context, loss to follow-up is defined as the discontinuation of medication use for 30 days or more. Primary loss to follow-up refers to individuals who either initiated treatment for less than 30 days and subsequently discontinued it for 30 days or more, or who never started the prescribed treatment [32].

The definition of the time frame was established from 2015 onwards, given the inclusion of the “special populations” field in the compulsory notification form in Brazil [33]. Cases identified with the “special populations” and/or “HIV” fields blank, unknown, result pending or not completed were excluded.

### Source of information, data collection and variables

This was a study based on secondary data from the Notifiable Diseases Information System (SINAN), regarding notifications of TB cases co-infected with HIV in the RHS in the municipality of São Paulo between 2015 and 2023. It should be noted that the 2023 data is provisional.

SINAN is a nationwide health information system that aims to collect, process, transmit and disseminate health information in a standardized way on diseases and illnesses that are compulsorily notifiable [33, 34]. The Tuberculosis Notification/Investigation and Follow-up Forms of the SINAN are completed after diagnosis and are subsequently updated and monitored throughout the individual’s treatment for TB, up to the treatment outcome, as well as being mandatory for notification in Brazil.

These forms are made available as open access data by the Department of Informatics of the Unified Health System (DATASUS). The data is anonymized in terms of personal information. The data was extracted on July 15, 2024, via RStudio using the download.file function and in.dbc format by downloading the URL for each year (2015–2023) available on the DATASUS “File Transfer” website. Since all the cases notified in the municipality during the established period were considered, there was no concern about the pairing of participants in the cohort.

The individual databases for each year were grouped together and checked for equal availability of variables. Duplicate notifications were identified and excluded using the distinct function. The coded variables, in the format in which they are available, were corrected for categories according to the data dictionary [35]. The continuous age variable was corrected and categorized into three age groups.

The variables that made up the study are of the nominal categorical type. For sociodemographic characterization, the following were considered: gender (female and male), race/color (white, brown, black, other, unknown), age group(15–29 years, 30–59 years and 60 years or more), education (0, 1 to 8 years and more than 8 years) and immigrant (yes and no). As for the clinical-epidemiological presentation, we considered: clinical presentation of TB (pulmonary, extrapulmonary and pulmonary + extrapulmonary), Type of TB case (new case, relapse and re-entry after loss to follow-up), associated diseases and conditions (diabetes, mental illness, alcoholism, smoking and AIDS), diagnostic sputum smear result (positive, negative and not performed), chest X-ray (normal, suspected, other pathology and not performed), sputum culture result (positive, negative, result pending and not performed), Xpert MTB/RIF (sensitive to Rifampicin, resistant to Rifampicin, inconclusive, not detectable and not carried out), drug susceptibility test (DST) result (sensitive, resistant to Isoniazid, resistant to Rifampicin, resistant to Isoniazid and Rifampicin, resistant to other drugs, result pending and not carried out), ART (yes, no and unknown), follow-up smears carried out (0–6), DOT (yes and no) and treatment outcome (cure, primary loss to follow-up and loss of follow-up).

After combining the years, cleaning and selecting the variables, the database was subjected to a filtering process through spatial delimitation followed by pre-established inclusion and exclusion criteria to cut out the study population.

### Statistical analysis

The study used the analysis software RStudio version 4.1.3 (Boston, MA, USA) and Jamovi 2.3.28 (Sydney, NSW, Australia), using the “tidyverse” and “read.dbc” packages for extracting, cleaning and selecting the variables, as well as delimiting the population and analyzing the data [36–39].

The cases were characterized using a descriptive analysis of the data with absolute and relative frequencies. At this stage, the universe of available data was analyzed, including information filled in as unknown, blank and/or without information.

To assess the outcomes of loss to follow-up in the defined study population, a multivariate analysis was carried out using binary logistic regression, reporting the Odds Ratio (OR) and its 95% confidence interval (95%CI). The assumption of collinearity was also analyzed using the variance inflation factor (VIF), as well as the model’s quality indicators using the Akaike information criterion (AIC), Nagelkerke’s pseudo R2 (R2N) and the area under the curve (AUC).

The binary analysis considered the following grouping of the dependent variable treatment outcome: loss to follow-up (loss to follow-up and primary loss to follow-up). The independent variables were: gender, race/color (white and non-white), age, schooling, immigrant, type of entry, clinical presentation of TB, associated diseases and conditions, sputum smear result(diagnosis), chest X-ray, sputum culture result, Xpert MTB/RIF, DST result, ART, control smear microscopies and DOT.

The model built used backward stepwise variable selection, in which the variables that showed *p* < 0.20 in the bivariate analysis were initially selected for the likelihood ratio test, followed by removal one by one until those with *p* < 0.05 and or with the best model indicators remained in the final model. In addition, the variables from the model for the homeless population were used for the general population model, given the subject of the study. The analysis script and the database have been provided as a supplementary file (File 1 and File 2).

## Results

**Fig. 1:**
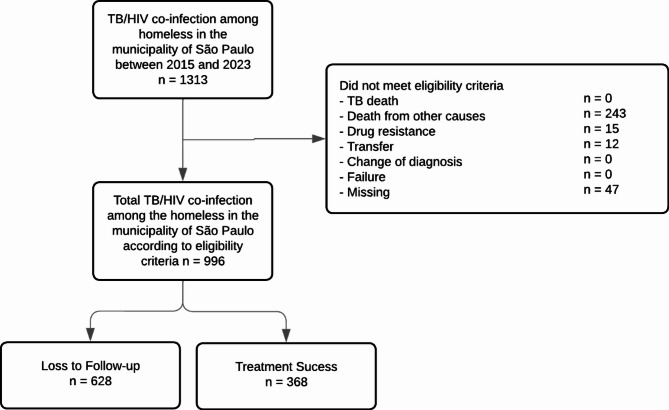
Flowchart of the selection process for the study population composed of people experiencing homelessness living with HIV who were lost to follow-up in tuberculosis treatment (*n* = 628) between 2015 and 2023 in the municipality of São Paulo

A total of 1,313 notifications of TB/HIV co-infection among homeless people in the municipality of São Paulo were identified between 2015 and 2023, and the outcomes that were not the subject of the study were removed (*n* = 317). Thus, 996 cases of TB with HIV co-infection among the homeless were analyzed, totaling 628 (63.05%) cases of loss of TB treatment follow-up. This process can be seen in Fig. 1.

Outcomes were led by loss of follow-up (*n* = 682; 47.15%), expressed by the variables “loss of follow-up” (*n* = 582; 43.59%) and “primary loss to follow-up” (*n* = 46; 3.45%), followed by “cure” (*n* = 368; 27.63%) and “death from other causes” (*n* = 248; 18.62%). The outcomes “death from TB” and “failure” were not filled in during the period studied, as well as some with no information (*n* = 47; 3.52%). The zero TB death can be explained by the overlap of HIV in relation to TB in the cause of death, being included in “death from other causes”.

For this study, cases of loss to follow-up were considered (*n* = 628; 63.05%). Regarding sociodemographic characteristics, most of the homeless population with TB/HIV co-infection were male (79.82%), non-migrants (94.68%), aged between 30 and 59 (76.81%), self-declared brown (43.67%) and with between one and eight years of schooling (40.76%), as shown in Table 1. A table of sociodemographic and clinical-epidemiological characteristics according to treatment outcomes (loss to follow-up and cure) stratified by population groups (homeless and general population) is disponible in splementaru file 3.

**Table 1:** Sociodemographic and clinical-epidemiological characteristics of TB-HIV coinfection cases reported by population group (homeless vs. general population), São paulo, 2015–2023

Characteristics	Homeless Population (*n* = 996)		General Population (*n* = 4.162)		Total (*n* = 5.158)
	*n*(%)	*p*-value*	*n*(%)	*p*-value*	*n*(%)
Sociodemographic characteristics					
Gender		0,303		< 0,001	
Female	201(20.18)		1138(27.34)		1339(25.96)
Male	795(79.82)		3024(72.66)		3819(74.04)
Race/Color		0,147		< 0,001	
White	297(29.82)		1568(37.67)		1865(36.16)
Brown	435(43.67)		1633(39.24)		2068(40.09)
Black	218(21.89)		635(15.26)		853(16.54)
Other	4(0.4)		28(0.67)		32(0.62)
Unknown	30(3.01)		195(4.69)		225(4.36)
Missing	12(1.2)		103(2.47)		115(2.23)
Age group (years)		< 0,064		< 0,001	
15 to 29	201(20.18)		989(23.76)		1190(23.07)
30 to 59	765(76.81)		2990(71.84)		3755(72.8)
≥ 60	30(3.01)		183(4.4)		213(4.13)
Years of schooling		0,729		0,001	
No schooling	25(2.51)		75(1.8)		100(1.94)
1 to 8 years	406(40.76)		1157(27.8)		1563(30.3)
More than 8 years	283(28.41)		2158(51.85)		2441(47.32)
Unknown	267(26.81)		641(15.4)		908(17.6)
Missing	15(1.51)		131(3.15)		146(2.83)
Migrant		0,803		0,029	
Yes	12(1.2)		78(1.87)		90(1.74)
No	943(94.68)		3882(93.27)		4825(93.54)
Missing	41(4.12)		202(4.85)		243(4.71)
Clinical conditions					
Clinical presentation of TB		0,368		< 0,001	
Pulmonary	838(84.14)		2700(64.87)		3538(68.59)
Extrapulmonary	63(6.33)		886(21.29)		949(18.4)
Pulmonary + Extrapulmonary	95(9.54)		576(13.84)		671(13.01)
Type of TB case		< 0,001		< 0,001	
New Case	443(44.48)		2775(66.67)		3218(62.39)
Recurrence	153(15.36)		607(14.58)		760(14.73)
Return after loss to follow up	400(40.16)		780(18.74)		1180(22.88)
Diabetes		0,123		0,048	
Yes	14(1.41)		79(1.9)		93(1.8)
No	982(98.59)		4083(98.1)		5065(98.2)
Mental Illness		0,621		0,324	
Yes	19(1.91)		76(1.83)		95(1.84)
No	977(98.09)		4086(98.17)		5063(98.16)
Alcoholism		0,195		< 0,001	
Yes	474(47.59)		725(17.42)		1199(23.25)
No	522(52.41)		3437(82.58)		3959(76.75)
Smoking		0,302		< 0,001	
Yes	345(34.64)		820(19.7)		1165(22.59)
No	651(65.36)		3342(80.3)		3993(77.41)
Illicit drug use		0,009		< 0,001	
Yes	655(65.76)		1025(24.63)		3478(67.43)
No	341(34.24)		3137(75.37)		1680(32.57)
Aids		0,337		0,018	
Yes	944(94.78)		3866(92.89)		4810(93.25)
No	52(5.22)		296(7.11)		348(6.75)
Use of antiretroviral therapy (ART)		< 0,001		< 0,001	
Yes	447(44.88)		2202(52.91)		2649(51.36)
No	159(15.96)		413(9.92)		572(11.09)
Unknown	66(6.63)		164(3.94)		230(4.46)
Missing	324(32.53)		1383(33.23)		1707(33.09)
Diagnostic tests					
Chest x-ray		0,685		< 0,001	
Normal	28(2.81)		482(11.58)		510(9.89)
Suspected	586(58.84)		2389(57.4)		2975(57.68)
Other pathology	9(0.9)		67(1.61)		76(1.47)
Not performed	373(37.45)		1224(29.41)		1597(30.96)
Sputum smear result		0,053		0,016	
Positive	298(29.92)		1092(26.24)		1390(26.95)
Negative	353(35.44)		1365(32.8)		1718(33.31)
Not performed	345(34.64)		1705(40.97)		2050(39.74)
Culture		0,021		0,2	
Positive	542(54.42)		1438(34.55)		1980(38.39)
Negative	147(14.76)		724(17.4)		871(16.89)
Result pending	10(1)		27(0.65)		37(0.72)
Not performed	296(29.72)		1952(46.9)		2248(43.58)
Missing	1(0.1)		21(0.5)		22(0.43)
Xpert MTB/RIF		0,209		0,237	
Detectable result sensitive to Rifampicin	521(52.31)		1522(36.57)		2043(39.61)
Detectable result resistant to Rifampicin	9(0.9)		42(1.01)		51(0.99)
Inconclusive	32(3.21)		84(2.02)		116(2.25)
Not detectable	143(14.36)		661(15.88)		804(15.59)
Not performed	262(26.31)		1585(38.08)		1847(35.81)
Missing	29(2.91)		268(6.44)		297(5.76)
Drug susceptibility test (DST) result		< 0,001		0,025	
Susceptible	434(43.57)		17(0.41)		1629(31.58)
Resistant only to Isoniazid only	16(1.61)		21(0.5)		37(0.72)
Resistant only to Rifampicin only	7(0.7)		1195(28.71)		24(0.47)
Resistant to isoniazid and rifampicin	0(0)		9(0.22)		9(0.17)
Resistant to other first line drugs	1(0.1)		4(0.1)		5(0.1)
Result pending	1(0.1)		1(0.02)		2(0.04)
Not done	537(53.92)		2915(70.04)		3452(66.93)
Missing	0(0)		0(0)		0(0)
Treatment and outcomes					
Directly observed treatment		< 0,001		< 0,001	
Yes	205(20.58)		684(16.43)		889(17.24)
No	466(46.79)		1377(33.09)		1843(35.73)
Ignored	325(32.63)		2101(50.48)		2426(47.03)
Number of follow-up sputum smear tests performed		< 0,001		< 0,001	
Not performed	314(31.53)		1634(39.26)		1948(37.77)
1 exam	164(16.47)		758(18.21)		922(17.88)
2 exams	101(10.14)		483(11.6)		584(11.32)
3 exams	87(8.73)		363(8.72)		450(8.72)
4 exams	55(5.52)		234(5.62)		289(5.6)
5 exams	42(4.22)		135(3.24)		177(3.43)
6 exams	30(3.01)		54(1.3)		84(1.63)
Missing	203(20.38)		501(12.04)		704(13.65)
Treatment outcome		*		*	
Treatment Success	368(36.95)		2796(67.18)		3164(61.34)
Primary loss to follow-up	46(4.62)		39(0.94)		85(1.65)
Loss to follow-up	582(58.43)		1327(31.88)		1909(37.01)

*Likelihood ratio test value from bivariate analysis with the dependent variable "treatment outcome”

Regarding clinical characteristics, the most prevalent clinical presentation of the disease was pulmonary TB (84.14%) and sensitive to Rifampicin by the Xpert MTB/RIF (52.31%) and DST result (43.57%). In addition, more than half of the sputum cultures results were positive (54.42%) and the chest X-rays were suspicious for TB (58.84%); however, sputum smear microscopies were more negative (35.44%). The main types of entry were new cases (44.48%), followed by re-entry after loss to follow-up (40.16%).

AIDS (94.78%) was present in most TB/HIV co-infection cases, although only 44.88% were using ART during TB treatment. Illicit drug use was also present in more than half of the cases (65.76%). Regarding medical conditions, the following stand out: Diabetes (1.41%), mental illness (1.91%), smoking (34.64%) and alcoholism (47.59%) were present in a minority of the population studied, however these percentages were higher than those observed in the general population.

TB diagnosis processes in the population included sputum smear microscopy (65.36%), sputum culture results (70.18%), Xpert MTB/RIF (71.78%), sensitivity tests (46.08%) and chest X-rays (62.55%).

As for the TB treatment follow-up process, the coverage of dose supervision by the DOT was 20.58%. In addition, sputum smear microscopy was offered during the six months of treatment, of which one test carried out during the period covered 16.47% of the cases, while six sputum smear microscopies only covered 3.01% of individuals; with 31.53% of the cases not having any tests carried out.

The sociodemographic and clinical-epidemiological characteristics were, for the most part, similar in proportion for both populations. However, the situation regarding the end of TB treatment showed opposite proportions, with 63.05% loss to follow-up for the homeless population and 67.18% cure for the general population.

**Fig. 2:**
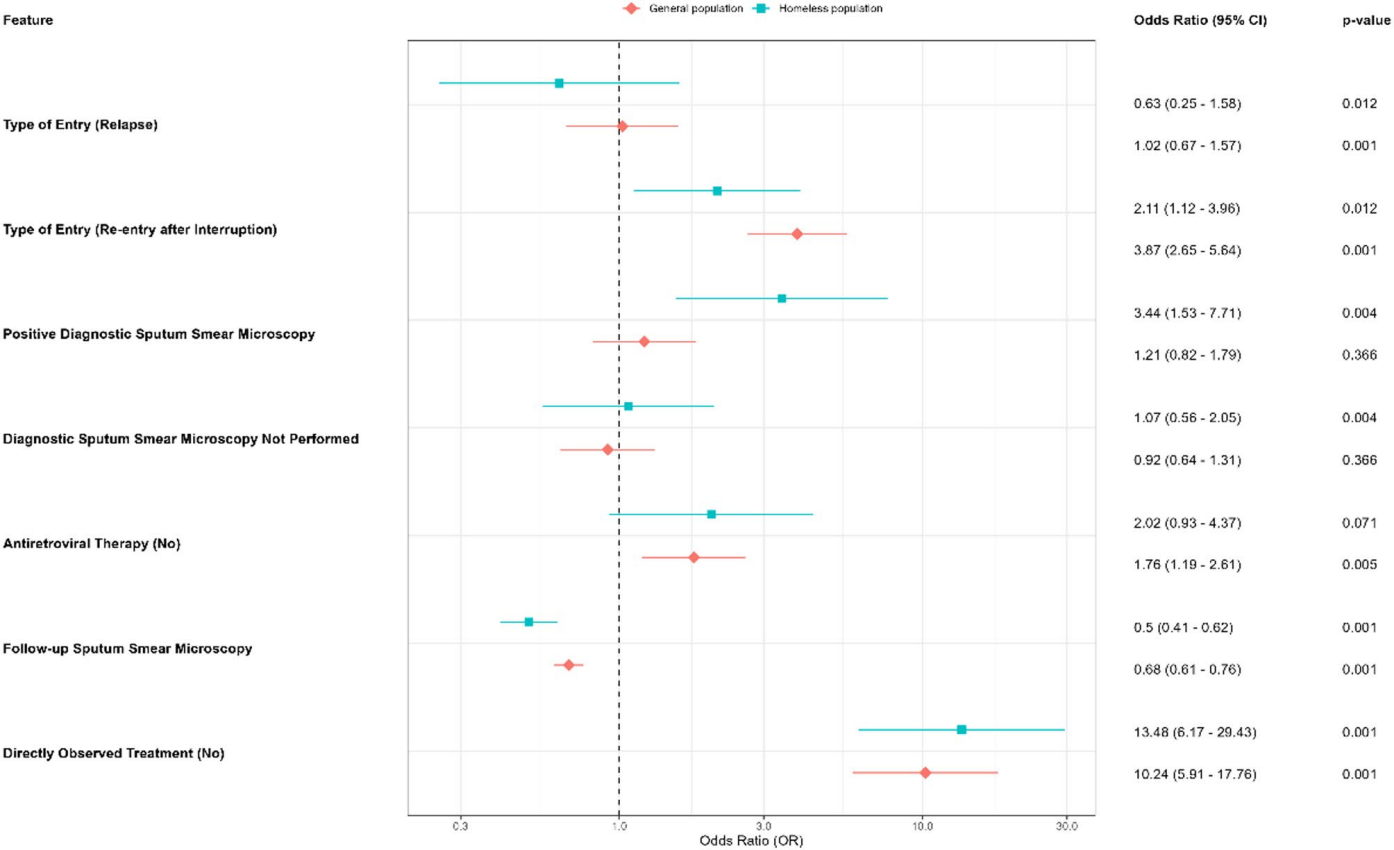
Forest plot of variables statistically significantly associated with loss to follow-up from tuberculosis treatment among people experiencing homelessness coinfected with HIV in São Paulo between 2015 and 2023

Legend: Forest plot of the adjusted logistic regression model. For the outcome variable (dependent), loss of follow-up was used as a reference. The references for the association variables were: new case for type of entry; yes for ART; negative for sputum smear result; and yes for directly observed treatment. Follow-up smear microscopy is discrete in nature, so it does not have a reference group.

For the initial association model, two sociodemographic variables and ten clinical-epidemiological variables were selected, as they had an established value of *p* < 0.20 (Table 1 and File 4). For the adjusted analysis of factors associated with an unfavorable outcome, loss to follow-up was significantly related to three clinical and epidemiological conditions (File 5) and its Forest plot in Fig. 2 shows the association.

It was observed that cases with treatment return after loss to follow-up (ORa = 2.10; 95%CI = 1.12–3.96) had a 110% greater chance of being lost to follow-up when compared to the cure outcome. A positive diagnostic smear test (ORa = 3.44; 95%CI = 1.53–7.71) was a risk factor for loss to follow-up, while having more follow-up smear tests (ORa = 0.52; 95%CI = 0.44–0.61) was a protective factor.

Homeless people who were not supervised by the DOT had a risk factor for loss to follow-up, with a 13.47 (95%CI = 6.17–29.42) greater chance of having this unfavorable outcome when compared to the cure outcome.

Similarly to what was identified for the homeless, the risk factors for loss to follow-up in the general population were returning to treatment (ORa = 3.86; 95%CI = 2.65–5.63) and not taking DOT (ORa = 10.24; 95%CI = 5.90-17.76), plus not use of ART (ORa = 1.76; 95%CI = 1.19–2.61). In addition, a higher number of follow-up sputum smear tests performed (ORa = 0.68; 95%CI = 0.61–0.76) was found to be a protective factor for loss to follow-up.

## Discussion

The homeless is a marginalized and socially vulnerable population segment that grows annually in Brazil, starting from 123 thousand people in this situation in 2015 and reaching 281 thousand people in 2022 [40]. In the country, the homeless have a 56 times greater risk of developing active TB than the general population [32]. Through this study, it was possible to verify a historical series of TB cases among homeless living with HIV in the city of São Paulo between 2015 and 2023 and their factors associated with loss of follow-up.

The time frame studied was marked mainly by the decrease in the cure rate and an increase in the loss of follow-up of individuals, especially from 2020 onwards, the time frame of the Covid-19 pandemic. The period exacerbated the vulnerability presented by the homeless through the increase in social inequality expressed by the difficulty in obtaining food, stigma and decreased access to health care, and socioeconomic conditions that were shaken during the period [41, 42].

Regarding sociodemographic characteristics, the population group studied was marked mainly by males, non-migrants, aged between 30 and 59 years, self-declared brown skin color, and who had between one and eight years of schooling. Studies developed seeking to understand the homeless and TB/HIV co-infection are in line with the variables gender, age, self-declared ethnic-racial identity, and schooling [9, 12, 43–45]. Furthermore, self-declaration of black and brown skin color, as well as lower levels of education were observed among homeless when compared to the general population, social characteristics of inequality, difficulty in accessing health care, and stigma, leading to a greater association with the development of active TB and unfavorable outcomes [9, 42].

During the pandemic period, there was a need to reorganize health care, directing efforts to the Covid-19 pandemic and leading to a dismantling of the actions offered for TB control, mainly in the search for respiratory symptoms and the implementation of DOT [10]. TB care in the city of São Paulo was initially suspended for three months, activities aimed at carrying out DOT and gradually the supervision of medication intake were resumed [20].

Providing DOT for homeless people as a healthcare strategy is essential to ensure support and supervision for individuals considering their social vulnerability and is considered the main strategy to ensure adherence to TB treatment, especially for people living with HIV. A national Brazilian study identified homeless people and HIV infection as the main risk factors for loss of follow-up of TB treatment [3]. In the city of São Paulo, statistical analysis identified that individuals not covered by DOT have a 13.47 (CI95 = 6.17–29.42) greater chance of loss of follow-up when compared to the outcome of cure. DOT helps with adherence to TB treatment, however, TB/HIV co-infection expresses a double journey of adherence that is essential for favorable outcomes for homeless people. Although the relevance of DOT for vulnerable populations, such as TB/HIV co-infected individuals experiencing homelessness, is particularly highlighted by the study, the findings may still be underestimated due to the high proportion of missing data within this variable.

The use of digital telecare strategies to provide care for people diagnosed with TB has proven effective among the general population ​ [46]​. The use of digital strategies, as a complement to health care, could result in the optimization of the work of the health team to adequately perform DOT for the most vulnerable populations, such as homeless people, especially those with HIV co-infection [47]​.

In the study population, although the association between loss to follow-up and non-adherence to use of ART was not statistically significant (ORa = 2.01; 95%CI = 0.92–4.3), the proportion of non-adherence to ART (15.96%) was significant. The lack of information (39.16%) may have contributed to the difficulty in establishing more reliable values ​​for the association. On the other hand, the non-significant values ​​were exposed after adjustment for confounding variables, which may indicate that it could be partially associated with another factor.

In addition to the aforementioned factor related to health care, the greater number of control sputum smear tests offered during the six months of treatment was considered a protective factor for loss to follow-up, a finding also found in another study [48]. This fact could suggest a greater connection between the user and the health system and adherence to the proposed treatment. Although this is a protective factor, the provision of sputum smear tests for homeless decreases drastically each month, covering only 3% in the sixth month of treatment. The use of sputum smear tests for diagnosing the disease is recommended in the municipality studied in cases of retreatment [49], a condition present in 55.5% of the notifications. Understanding that a positive bacilloscopy indicates bacilliferous cases, the association found in the study highlights a worrying context in the perpetuation of the chain of transmission of the disease [32], given the increased risk of loss to follow-up, by those with a positive test. Furthermore, this may be another indication of the vulnerability that involves the individual in a scenario of homelessness and positive for HIV.

Loss to follow-up is a serious event that not only causes the disease to continue spreading, especially among homeless people due to their social vulnerability and lifestyle but could also lead to an increased vulnerability to the development of drug resistance. Furthermore, the loss to follow-up indicator is directly related to re-entry into treatment, which has been shown to be a factor of vulnerability to loss to follow-up in the population. Individuals who are lost to follow-up for TB treatment once are 2.10 times more likely to lose to follow-up again when compared to those who are cured.

Although previous studies have shown that alcoholism, smoking, and illicit drug use are factors that make them vulnerable to an unfavorable outcome of TB and justified the inclusion of these variables in the final model, the sociodemographic characteristics presented by the group studied were not shown to be factors associated with loss to follow-up through statistical analyses [3, 43]. However, the frequency of use and abuse of these substances was considerably higher among homeless people when compared to the general population in the city of São Paulo.

Therefore, knowing the factors associated with loss to follow-up expressed in sociodemographic, clinical and health care factors can help in designing health actions and strategies to be offered to the population. In this study, failure to perform DOT was the main factor associated with loss to follow-up of homeless people in follow-up with TB/HIV co-infection and, although recommended for all homeless people, coverage was only for approximately 20% of cases. The limitations of the study include the erroneous completion and incompleteness of the TB notification forms, which account for a large percentage of the data and result in the exclusion of information, making it impossible to conduct analyses that are closer to reality, which may have mainly influenced the values ​​inferred about the DOT and use of ART. Despite the limitations mentioned, the study can contribute to providing support for understanding the magnitude of TB/HIV co-infection in homeless people according to loss to follow-up.

## Conclusion

The factors associated with loss to TB follow-up among homeless people living with HIV were Type of TB case, diagnostic and control bacilloscopy, and completion of DOT, which are primarily related to the organization of care and patient-provider engagement. The main protective factor related to loss to follow-up was DOT, therefore, its provision and the strengthening of supervision strategies focused on the needs of homeless people are essential to improve the indicators of loss to follow-up among homeless people who are treated for TB/HIV co-infection.

## Supplementary Information

Supplementary Material 1.

Supplementary Material 2.

Supplementary Material 3.

Supplementary Material 4.

Supplementary Material 5.

## Data Availability

Data and code are available in supplementary materials. The open data are available at https:/datasus.saude.gov.br/informacoes-de-saude-tabnet.

## Abbreviations

TB: Tuberculosis
HIV: Human immunodeficiency virus
DOT: Directly observed treatment
SINAN: Notifiable diseases information system
DATASUS: Department of informatics of the unified health system
DST: drug susceptibility test
OR: Odds ratio
Ora: Adjusted odds ratio
VIF: Variance inflation factor
AIC: Akaike information criterion
RN2: Nagelkerke’s pseudo R2
AUC: Area under the curve
ART: Antiretroviral therapy
