# Supplementary material for: TB/HIV co-infection in homelessness and factors associated with loss to follow-up of tuberculosis treatment: a retrospective cohort
Source: BMC Infect Dis. 2025 Sep 26;25:1158. doi: 10.1186/s12879-025-11532-y (PMC12466028; doi:10.1186/s12879-025-11532-y)
Supplement: Supplementary file 3 — Supplementary Material 3. [file 12879_2025_11532_MOESM3_ESM.docx]

# **File 4 Association between homeless and general populations and loss to follow-up in TB-HIV coinfection cases, unadjusted in the logistic regression model, São Paulo, 2015-2023.**

| **Characteristics** | **Loss of follow-up for**  **homeless population** | | **Loss to follow-up for the general population** | |
| --- | --- | --- | --- | --- |
|  | **OR(IC95%)** | **p-value*** | **OR(IC95%)** | **p-value*** |
| **Gender** |  | 0,303 |  | **< 0,001** |
| Female | 1.19(0.85-1.64) |  | 1.35(1.17-1.56) |  |
| Male | reference |  | reference |  |
| **Race/Color** |  | **0,147** |  | **< 0,001** |
| White | reference |  | reference |  |
| No white | 1.23(0.93-1.63) |  | 1.34(1.17-1.54) |  |
| **Age group (years)** |  | **< 0,064** |  | **< 0,001** |
| 15 to 59 | 1.99(0.96-4.13) |  | 2.29(1.56-3.35) |  |
| ≥ 60 | reference |  | reference |  |
| **Years of schooling** |  | 0,729 |  | **0,001** |
| No schooling | 1.29(0.54-3.05) |  | 0.89(0.54-1.45) |  |
| 1 to 8 years | reference |  | reference |  |
| More than 8 years | 1.11(0.80-1.52) |  | 0.75(0.65-0.87) |  |
| **Migrant** |  | 0,803 |  | **0,029** |
| Yes | 1.16(0.34-3.9) |  | 1.66(1.06-2.61) |  |
| No | reference |  | reference |  |
| **Clinical presentation of TB** |  | 0,368 |  | **< 0,001** |
| Pulmonary | reference |  | reference |  |
| Extrapulmonary | 1.41(0.80-2.46) |  | 0.60(0.50-0.71) |  |
| Pulmonary + Extrapulmonary | 1.2(0.76-1.87) |  | 0.77(0.63-0.93) |  |
| **Type of TB case** |  | **< 0,001** |  | **< 0,001** |
| New Case | reference |  | reference |  |
| Recurrence | 0.72(0.50-1.05) |  | 1.39(1.14-1.68) |  |
| Return after loss to follow up | 1.47(1.108-1.96) |  | 4.83(4.08-5.71) |  |
| **Diabetes** |  | **0,123** |  | **0,048** |
| Yes | 0.43(0.14-1.26) |  | 0.59(0.35-1.01) |  |
| No | reference |  | reference |  |
| **Mental Illness** |  | 0,621 |  | 0,324 |
| Yes | 1.28(0.48-3.38) |  | 0.77(0.46-1.29) |  |
| No | reference |  | reference |  |
| **Alcoholism** |  | **0,195** |  | **< 0,001** |
| Yes | 0.84(0.65-1.09) |  | 1.50(1.27-1.77) |  |
| No | reference |  | reference |  |
| **Smoking** |  | 0,302 |  | **< 0,001** |
| Yes | 1.15(0.87-1.51) |  | 1.48(1.27-1.74) |  |
| No | reference |  | reference |  |
| **Illicit drug use** |  | **0,009** |  | **< 0,001** |
| Yes | 1.43(1.1-1.87) |  | 2.947(2.54-3.41) |  |
| No | reference |  | reference |  |
| **Aids** |  | 0,337 |  | **0,018** |
| Yes | 0.74(0.40-1.37) |  | 1.37(1.05-1.78) |  |
| No | reference |  | reference |  |
| **Use of antiretroviral therapy (ART)** |  | **< 0,001** |  | **< 0,001** |
| Yes | reference |  | reference |  |
| No | 2.9(1.91-4.37) |  | 2.77(2.23-3.43) |  |
| **Chest x-ray** |  | 0,685 |  | **< 0,001** |
| Normal | 1.42(0.61-3.3) |  | 0.63(0.50-0.8) |  |
| Suspected | reference |  | reference |  |
| Other pathology | 0.71(0.19-2.69) |  | 1.13(0.68-1.88) |  |
| Not performed | 0.91(0.70-1.2) |  | 1.14(0.98-1.32) |  |
| **Sputum smear microscopy** |  | **0,053** |  | **0,016** |
| Positive | 1.48(1.06-2.04) |  | 1.005(0.85-1.18) |  |
| Negative | reference |  | reference |  |
| Not performed | 1.11(0.82-1.51) |  | 0.82(0.71-0.96) |  |
| **Culture** |  | **0,021** |  | 0,2 |
| Positive | 1.23(0.84-1.78) |  | 1.18(0.97-1.43) |  |
| Negative | reference |  | reference |  |
| Not performed | 1.71(1.13-2.57) |  | 1.07(0.89-1.29) |  |
| **Xpert MTB/RIF** |  | 0,209 |  | 0,237 |
| Detectable | 1.07(0.73-1.58) |  | 1.08(0.89-1.316) |  |
| Inconclusive | 0.64(0.29-1.39) |  | 1.15(0.71-1.85) |  |
| Not detectable | reference |  | reference |  |
| Not performed | 1.31(0.86-2.01) |  | 0.934(0.76-1.13) |  |
| **Sensitivity test** |  | **< 0,001** |  | **0,025** |
| Sensitive | reference |  | reference |  |
| Resistant | 3.97(1.33-11.8) |  | 2.188(1.24-3.83) |  |
| Not performed | 1.7(1.31-2.21) |  | 1.02(0.88-1.18) |  |
| **Directly Observed Treatment** |  | **< 0,001** |  | **< 0,001** |
| Yes | reference |  | reference |  |
| No | 22.44(13.57-37.10) |  | 15.94(10.54-24.12) |  |
| **Follow-up sputum smear microscopy performed** |  | **< 0,001** |  | **< 0,001** |
| 0-6 | 0.44(0.39-0.50) |  | 0.65(0.61-0.69) |  |
