# Supplementary material for: TB/HIV co-infection in homelessness and factors associated with loss to follow-up of tuberculosis treatment: a retrospective cohort
Source: BMC Infect Dis. 2025 Sep 26;25:1158. doi: 10.1186/s12879-025-11532-y (PMC12466028; doi:10.1186/s12879-025-11532-y)
Supplement: Supplementary file 4 — Supplementary Material 4. [file 12879_2025_11532_MOESM4_ESM.rtf]

File 5	 Association between homeless and general populations and loss to follow-up in TB-HIV coinfection cases, adjusted for sociodemographic and clinical-epidemiological variables in the logistic regression model.
Characteristics 	Loss of follow-up for homeless population†	Loss to follow-up for the general population‡	
 	ORa(IC95)	p-value*	ORa(IC95)	p-value*	
Type of Admission	 	0,012	 	< 0,001	 	
New Case	reference	 	reference	 	 	
Return after loss to follow up/interruption	2.10(1.12-3.96)	 	3.86(2.65-5.63)	 	 	
 Recurrence	0.63(0.25-1.58)	 	1.02(0.66-1.56)	 	 	
Antiretroviral therapy	 	0,070	 	0,005	 	
Yes	reference	 	reference	 	 	
No	2.01(0.92-4.3)	 	1.76(1.19-2.61)	 	 	
Sputum smear microscopy	 	0,004	 	0,366	 	
 Positive	3.44(1.53-7.71)	 	1.21(0.81-1.79)	 	 	
 Negative	reference	 	reference	 	 	
 Not performed	1.073(0.56-2.054)	 	0.91(0.64-1.31)	 	 	
Follow-up sputum smear microscopy performed	 	< 0,001	 	< 0,001	 	
 0-6	0.50(0.40-0.62)	 	0.68(0.61-0.76)	 	 	
Directly observed treatment	 	< 0,001	 	< 0,001	 	
 Yes	reference	 	reference	 	 	
 No	13.47(6.17-29.42)	 	10.24(5.90-17.76)	 	 	
Legenda: *P value by the likelihood ratio test. †Model indicators: VIF: 1.01 to 1.05; AIC: 320; pseudo R2N: 0.552; AUC: 0.891. ‡Model indicators: VIF: 1.002 to 1.032; AIC: 1054; pseudo R2N: 0.308; AUC: 0.769.
