# Supplementary material for: TB/HIV co-infection in homelessness and factors associated with loss to follow-up of tuberculosis treatment: a retrospective cohort
Source: BMC Infect Dis. 2025 Sep 26;25:1158. doi: 10.1186/s12879-025-11532-y (PMC12466028; doi:10.1186/s12879-025-11532-y)
Supplement: Supplementary file 5 — Supplementary Material 5. [file 12879_2025_11532_MOESM5_ESM.docx]

# **File 3 Sociodemographic and clinical-epidemiological characteristics of TB-HIV coinfection cases reported by population group (homeless and general population) and treatment outcome (treatment success and loss to follow-up), São Paulo, 2015-2023**

| **Characteristics** | **Homeless Population** | | **General Population** | |
| --- | --- | --- | --- | --- |
|  | **Treatment Success** | **Loss to follow-up** | **Treatment Success** | **Loss to follow-up** |
|  | **n(%)** | **n(%)** | **n(%)** | **n(%)** |
| **Gender** |  |  |  |  |
| Female | 68(6.83) | 133(13.35) | 708(17.01) | 430(10.33) |
| Male | 300(30.12) | 495(49.7) | 2088(50.17) | 936(22.49) |
| **Race/Color** |  |  |  |  |
| White | 119(11.95) | 178(17.87) | 1105(26.55) | 463(11.12) |
| Brown | 158(15.86) | 277(27.81) | 1049(25.2) | 584(14.03) |
| Black | 73(7.33) | 145(14.56) | 402(9.66) | 233(5.6) |
| Other | 0(0) | 4(0.4) | 18(0.43) | 10(0.24) |
| Unknown | 13(1.31) | 17(1.71) | 146(3.51) | 49(1.18) |
| Missing | 5(0.5) | 7(0.7) | 76(1.83) | 27(0.65) |
| **Age group (years)** |  |  |  |  |
| 15 to 29 | 65(6.53) | 136(13.65) | 598(14.37) | 391(9.39) |
| 30 to 59 | 287(28.82) | 478(47.99) | 2048(49.21) | 942(22.63) |
| ≥ 60 | 16(1.61) | 14(1.41) | 150(3.6) | 33(0.79) |
| **Years of schooling** |  |  |  |  |
| No schooling | 8(0.8) | 17(1.71) | 49(1.18) | 26(0.62) |
| 1 to 8 years | 153(15.36) | 253(25.4) | 726(17.44) | 431(10.36) |
| More than 8 years | 100(10.04) | 183(18.37) | 1489(35.78) | 669(16.07) |
| Unknown | 104(10.44) | 163(16.37) | 435(10.45) | 206(4.95) |
| Missing | 3(0.3) | 12(1.2) | 97(2.33) | 34(0.82) |
| **Migrant** |  |  |  |  |
| Yes | 4(0.4) | 8(0.8) | 43(1.03) | 35(0.84) |
| No | 347(34.84) | 596(59.84) | 2608(62.66) | 1274(30.61) |
| Missing | 17(1.71) | 24(2.41) | 145(3.48) | 57(1.37) |
| **Clinical presentation of TB** |  |  |  |  |
| Pulmonary | 317(31.83) | 521(52.31) | 1731(41.59) | 969(23.28) |
| Extrapulmonary | 19(1.91) | 44(4.42) | 663(15.93) | 223(5.36) |
| Pulmonary + Extrapulmonary | 32(3.21) | 63(6.33) | 402(9.66) | 174(4.18) |
| **Type of TB case** |  |  |  |  |
| New Case | 174(17.47) | 269(27.01) | 2082(50.02) | 693(16.65) |
| Recurrence | 72(7.23) | 81(8.13) | 415(9.97) | 192(4.61) |
| Return after loss to follow up | 122(12.25) | 278(27.91) | 299(7.18) | 481(11.56) |
| **Diabetes** |  |  |  |  |
| Yes | 8(0.8) | 6(0.6) | 61(1.47) | 18(0.43) |
| No | 360(36.14) | 622(62.45) | 2735(65.71) | 1348(32.39) |
| **Mental Illness** |  |  |  |  |
| Yes | 6(0.6) | 13(1.31) | 55(1.32) | 21(0.5) |
| No | 362(36.35) | 615(61.75) | 2741(65.86) | 1345(32.32) |
| **Alcoholism** |  |  |  |  |
| Yes | 185(18.57) | 289(29.02) | 431(10.36) | 294(7.06) |
| No | 183(18.37) | 339(34.04) | 2365(56.82) | 1072(25.76) |
| **Smoking** |  |  |  |  |
| Yes | 120(12.05) | 225(22.59) | 491(11.8) | 329(7.9) |
| No | 248(24.9) | 403(40.46) | 2305(55.38) | 1037(24.92) |
| **Illicit drug use** |  |  |  |  |
| Yes | 223(22.39) | 432(43.37) | 495(11.89) | 530(12.73) |
| No | 145(14.56) | 196(19.68) | 2301(55.29) | 836(20.09) |
| **Aids** |  |  |  |  |
| Yes | 352(35.34) | 592(59.44) | 2579(61.97) | 1287(30.92) |
| No | 16(1.61) | 36(3.61) | 217(5.21) | 79(1.9) |
| **Use of antiretroviral therapy (ART)** |  |  |  |  |
| Yes | 209(20.98) | 238(23.9) | 1560(37.48) | 642(15.43) |
| No | 37(3.71) | 122(12.25) | 193(4.64) | 220(5.29) |
| Unknown | 22(2.21) | 44(4.42) | 104(2.5) | 60(1.44) |
| Missing | 100(10.04) | 224(22.49) | 939(22.56) | 444(10.67) |
| **Chest x-ray** |  |  |  |  |
| Normal | 8(0.8) | 20(2.01) | 367(8.82) | 115(2.76) |
| Suspected | 213(21.39) | 373(37.45) | 1602(38.49) | 787(18.91) |
| Other pathology | 4(0.4) | 5(0.5) | 43(1.03) | 24(0.58) |
| Not performed | 143(14.36) | 230(23.09) | 784(18.84) | 440(10.57) |
| **Sputum smear microscopy** |  |  |  |  |
| Positive | 94(9.44) | 204(20.48) | 714(17.16) | 378(9.08) |
| Negative | 143(14.36) | 210(21.08) | 894(21.48) | 471(11.32) |
| Not performed | 131(13.15) | 214(21.49) | 1188(28.54) | 517(12.42) |
| **Culture** |  |  |  |  |
| Positive | 209(20.98) | 333(33.43) | 947(22.75) | 491(11.8) |
| Negative | 64(6.43) | 83(8.33) | 503(12.09) | 221(5.31) |
| In progress | 2(0.2) | 8(0.8) | 8(0.19) | 19(0.46) |
| Not performed | 92(9.24) | 204(20.48) | 1326(31.86) | 626(15.04) |
| Missing | 1(0.1) | 0(0) | 12(0.29) | 9(0.22) |
| **Xpert MTB/RIF** |  |  |  |  |
| Detectable result sensitive to Rifampicin | 198(19.88) | 323(32.43) | 1007(24.2) | 515(12.37) |
| Detectable result resistant to Rifampicin | 0(0) | 9(0.9) | 20(0.48) | 22(0.53) |
| Inconclusive | 16(1.61) | 16(1.61) | 54(1.3) | 30(0.72) |
| Not detectable | 56(5.62) | 87(8.73) | 446(10.72) | 215(5.17) |
| Not performed | 86(8.63) | 176(17.67) | 1093(26.26) | 492(11.82) |
| Missing | 12(1.2) | 17(1.71) | 176(4.23) | 92(2.21) |
| **Sensitivity test** |  |  |  |  |
| Sensitive | 192(19.28) | 242(24.3) | 810(19.46) | 385(9.25) |
| Resistant only to Isoniazid | 3(0.3) | 13(1.31) | 12(0.29) | 9(0.22) |
| Resistant only to Rifampicin | 1(0.1) | 6(0.6) | 8(0.19) | 9(0.22) |
| Resistant to Isoniazida and Rifampicina | 0(0) | 0(0) | 3(0.07) | 6(0.14) |
| Resistant to other 1st line drugs | 0(0) | 1(0.1) | 2(0.05) | 2(0.05) |
| In progress | 1(0.1) | 0(0) | 0(0) | 1(0.02) |
| Not performed | 171(17.17) | 366(36.75) | 1961(47.12) | 954(22.92) |
| Missing | 0(0) | 0(0) | 0(0) | 0(0) |
| **Directly Observed Treatment** |  |  |  |  |
| Yes | 185(18.57) | 20(2.01) | 659(15.83) | 25(0.6) |
| No | 136(13.65) | 330(33.13) | 858(20.62) | 519(12.47) |
| Ignored | 47(4.72) | 278(27.91) | 1279(30.73) | 822(19.75) |
| **Follow-up sputum smear microscopy performed** |  |  |  |  |
| Not performed | 50(5.02) | 389(39.06) | 1117(26.84) | 865(20.78) |
| 1 exam | 74(7.43) | 133(13.35) | 557(13.38) | 302(7.26) |
| 2 exams | 63(6.33) | 52(5.22) | 381(9.15) | 133(3.2) |
| 3 exams | 63(6.33) | 34(3.41) | 341(8.19) | 35(0.84) |
| 4 exams | 48(4.82) | 12(1.2) | 221(5.31) | 18(0.43) |
| 5 exams | 45(4.52) | 3(0.3) | 127(3.05) | 11(0.26) |
| 6 exams | 25(2.51) | 5(0.5) | 52(1.25) | 2(0.05) |
